# Reply to ‘Stability and compatibility of antibiotics in PD solutions—call for including antibiotics for drug-resistant infections’

**DOI:** 10.1093/ckj/sfac132

**Published:** 2022-05-19

**Authors:** David W Johnson, Simon Wai Yin So, Philip Kam-Tao Li

**Affiliations:** University of Queensland at Princess Alexandra Hospital, Brisbane, QLD, Australia; Pharmacy Department, Alice Ho Miu Ling Nethersole Hospital, Tai Po, Hong Kong; Department of Medicine and Therapeutics, Carol and Richard Yu Peritoneal Dialysis Research Centre, Prince of Wales Hospital, Chinese University of Hong Kong, Shatin, Hong Kong

We thank Ling *et al*. for their letter [1] regarding our article [2].

While we attempted to provide a comprehensive review of recent stability and compatibility studies pertaining to a wide range of antibiotics admixed in various peritoneal dialysis (PD) solutions, we freely acknowledged in the article that there was a ‘paucity of stability and compatibility data of antibiotics in PD solutions’. We further emphasized in our concluding remarks that ‘knowledge gaps still exist regarding the impact of administration of antibiotics in different compartments of dual-chamber bags and the stability and compatibility data of antifungal agents and narrow-spectrum antibiotics targeting specific species’ and that ‘future research is required in this area’ [2].

We agree with Ling *et al*. [1] that an updated review that summarizes the chemical stability and physical compatibilities of newer antibiotics in peritoneal dialysis solutions would be useful and we would welcome studies in this area to permit such an update to be performed in the future.
